# Epidemiological Trends in Pediatric Osteoarticular Infections—Results from a Single-Center Retrospective Study Covering 2015–2023

**DOI:** 10.3390/children12091210

**Published:** 2025-09-10

**Authors:** Evelien B. van Kempen, Ayla Scholma, Nam Nam Cheung, Mirjam van Veen, Joost H. van Linge

**Affiliations:** 1 Department of Pediatrics, Juliana Children’s Hospital Haga Hospital, 2509 AB The Hague, The Netherlands; 2 Department of Medical Microbiology, Haga Hospital, 2509 AB The Hague, The Netherlands; 3 Department of Orthopedic Surgery, Juliana Children’s Hospital Haga Hospital, 2545 AA The Hague, The Netherlands; j.h.vanlinge@hagaziekenhuis.nl

**Keywords:** osteoarticular infection, *Kingella kingae*, Group A streptococcus, epidemiology, post-COVID-19 pandemic

## Abstract

**Highlights:**

**What are the main findings?**

- In our observational single-center study, pediatric osteoarticular infection increased from 10/year between 2015 and 2019 to 21/year between 2022 and 2023.
- During the pandemic period, a non-significant decrease in *Kingella kingae* and Group A streptococcus was observed, accompanied by a relative increase in *Staphylococcus aureus*. In contrast, the post-pandemic period showed a proportional rise in *Kingella kingae* detections, while the contribution of *Staphylococcus aureus* declined.

**What is the implication of the main finding?**

- Post-pandemic, there seems to be an increase in pediatric osteoarticular infections. This highlights the need for further multicenter research to assess whether this reflects a broader epidemiological change and to guide future diagnostic and therapeutic strategies.

**Abstract:**

Background: Pediatric infectious disease epidemiology has changed since the COVID-19 pandemic. To investigate possible changes in the epidemiology of pediatric osteoarticular infections (pOAIs), entailing osteomyelitis (OM), septic or infectious arthritis (AR), and osteomyelitis combined with arthritis (OA), we aimed to assess the number of pOAI cases, pathogen distribution, and outcomes across the pre-, mid-, and post-pandemic periods. Methods: We conducted a single-center retrospective cohort study in the Dutch Juliana Children’s Hospital, including patients aged 0–18 years diagnosed with OM, AR, or OA between 2015 and 2023. Cases were grouped into three periods: pre-pandemic (P1: 2015–2019), mid-pandemic (P2: 2020–2021), and post-pandemic (P3: 2022–2023). Data on demographics, clinical course, imaging, microbiology, and outcomes were extracted from medical records. Results: A total of 118 pOAI cases (median age 2 years, IQR 1–8) were included. OM occurred in 50%, AR in 42%, and OA in 8% of cases. Annual case counts increased from an average of 10/year in P1 to 21/year in P3. Although the difference between P1 and P2 was not statistically significant (IRR 1.20; 95% CI 0.70–2.06), there was a significant increase in P3 compared to P1 (IRR 1.97; 95% CI 1.31–2.97). Pathogen detection was achieved in 50% of cases. *Staphylococcus aureus* remained the most frequently identified pathogen overall. From P1 to P2, proportions of *Kingella kingae* and GAS declined, while *Staphylococcus aureus* remained stable. In P3, *Kingella kingae* increased, *Staphylococcus aureus* decreased, and GAS remained relatively unchanged. However, none of these changes were statistically significant. No patients required PICU admission or experienced fatal outcomes. Conclusion: This study suggests an increase in pOAI after the COVID-19 pandemic. While patient characteristics and outcome remained similar over time, pathogen distribution seems to have changed throughout the periods.

## 1. Introduction

The epidemiology of pediatric infectious diseases has shifted markedly in the wake of the COVID-19 pandemic and the widespread implementation of non-pharmaceutical interventions (NPIs) aimed at curbing SARS-CoV-2 transmission [1,2,3]. During the period in which NPIs were enforced, numerous countries reported substantial declines in the incidence of common pediatric communicable diseases, particularly those transmitted via airborne routes. However, a resurgence of infectious diseases has been observed following the relaxation of these measures [1,2,3].

Pediatric osteoarticular infections (pOAIs), comprising osteomyelitis (OM), septic or infectious arthritis (AR), and combined osteomyelitis with arthritis (OA), typically arise via hematogenous spread, wherein bacteria from colonized mucosal or skin surfaces enter the bloodstream and infiltrate bone or joint tissue [4,5,6]. In children, this route accounts for the vast majority of OAI cases, particularly in the metaphyseal regions of long bones due to their rich vascular supply. Minor trauma and viral infections may further facilitate bacterial translocation through the disruption of local vasculature or epithelial barriers. The most common causative organisms in pOAI are *Staphylococcus aureus* and *Kingella kingae* in children younger than 5 years [6]. Other pathogens are *Streptococcus pyogenes* (also referred to as Group A streptococcus, or GAS), *Streptococcus pneumonia*, and *Haemophilus influenzae* type b [4,5,6].

While several studies have examined the impact of the pandemic on invasive bacterial infections in children, little is known about its influence on the incidence of pOAIs. Given the documented post-pandemic rise in respiratory viral infections, concurrent increases in bacterial infections like GAS, and the potential for increased individual susceptibility, we hypothesized a corresponding increase in the incidence of pOAIs [7,8,9,10]. To our knowledge, this has not yet been systematically investigated. In this study, we aimed to evaluate the epidemiology of pOAIs in the pre- (2015–2019), mid- (2020–2021), and post-COVID-19 (2022–2023) pandemic.

## 2. Materials and Methods

We conducted a retrospective cohort study at Juliana Children’s Hospital Haga Hospital, a large regional teaching hospital in the city of The Hague, The Netherlands. The hospital provides general pediatric care to the local urban population as well as specialized care—such as pediatric surgery and pediatric orthopedics—for referred patients from the wider region. The study was approved by the medical ethical review committee. Need for informed consent was waived.

### 2.1. Participants

Children aged 0–18 years diagnosed with OM, AR, or OA, as defined by the clinical criteria established by Trobisch et al., presenting between January 2015 and December 2023, were eligible for inclusion (Table 1, Supplementary Materials) [4]. Exclusion criteria entailed non-infectious-related causes of pOAI (including reactive origins) and hospitalization elsewhere.

### 2.2. Data Collection

Eligible cases were identified using CTcue^®^ software (www.ctcue.com, accessed between 1 September 2023 and 31 January 2024 ), a clinical data extraction tool. All identified cases were reviewed for eligibility based on the inclusion and exclusion criteria. Clinical data were extracted from electronic medical records and included patient demographics, diagnosis and anatomical site of infection, diagnostics, illness severity (pediatric intensive care unit admission, mortality), treatment, and surgical procedures and complications. Needle puncture and drainage alone were not classified as surgical procedures. Data were compared across the pre- (2015–2019, period 1), mid- (2020–2021, period 2), and post- (2022–2023, period 3) COVID-19 pandemic periods. In The Netherlands, NPIs were gradually phased out starting in January 2022, with all major restrictions lifted by March 2022 [11].

### 2.3. Analysis

As this was a retrospective study using available clinical data, no power calculation was performed. Descriptive statistics were used to summarize baseline characteristics. Continuous variables are reported as mean ± standard deviation (SD) or median with interquartile range (IQR). Categorical variables are presented as counts and percentages. Group comparisons were performed using the *t*-test, Mann–Whitney U test, and Fisher’s exact test or chi-squared test. A two-sided *p* value < 0.05 was considered statistically significant. All analyses were performed and visualized using RStudio (version 4.3.1).

## 3. Results

**Figure 1:**
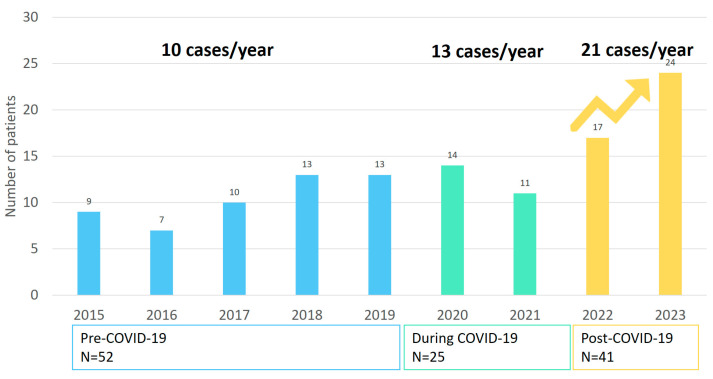
Number of patients diagnosed with pediatric osteoarticular infections per year between 2015 and 2023.

After screening 588 eligible patients, 118 pOAI cases were included, and 59/118 (50%) cases had OM, 50/118 (42.4%) AR, and 9/118 (7.6%) OA (Figure 1, Table 2). Median age was 2 years (IQR 1–8). No patients had sepsis at presentation or during hospitalization. Admission to pediatric intensive care unit did not occur. None of the cases had a fatal outcome.

### 3.1. Basic Characteristics Regarding Pre-, Mid-, and Post-COVID-19 Pandemic Periods

The annual number of pOAI cases increased from 9 in 2015 to 24 in 2023 (Figure 1). In P1, 52 pOAI cases were diagnosed, averaging 10 cases/year. In P2, 25 cases were identified, an average of 13 cases/year. In P3, this increased to 41 cases, yielding 21 cases/year. There was no statistically significant difference in the annual case rate between the pre-pandemic and pandemic period, with an incidence rate ratio (IRR) of 1.20 (95% CI 0.70–2.06). The average annual number of cases in P3 increased by 56.7% compared to P1, corresponding to an incidence rate ratio (IRR) of 1.97 (95% CI, 1.31–2.97). Age, gender, and diagnosis did not statistically differ across the three time periods (Table 1).

### 3.2. Diagnostic Findings

In total, 71/118 (60%) joint and/or bone punctures were performed. The annual number of punctures conducted increased over time (Table 3). Molecular detection of *Kingella kingae* by PCR was introduced in 2017, with testing performed in 12.5% of cases that year. Utilization increased to 60% by 2019, followed by a decline in 2020. From 2021 to 2023, PCR testing rates stabilized (Table 3).

An abscess was identified at presentation in 23 of 118 patients (19.5%). Of these, 18/23 (78%) had OM, 4/23 (17%) AR, and 1/23 (4%) OA. The proportion of patients presenting with an abscess increased over time: 5/51 (10%) cases in P1, 7/25 (28%) in P2, and 11/41 (26.8%) in P3. Among patients with an abscess, imaging modalities included MRI in 15/23 (65%) cases, ultrasound in 6/23 (26%), PET-CT in 1/23 (4%), and X-ray in 1/23 (4%). MRI was performed in 43/118 cases, with 19 in P1, 9 in P2, and 15 in P3.

### 3.3. Pathogen Distribution

**Figure 2:**
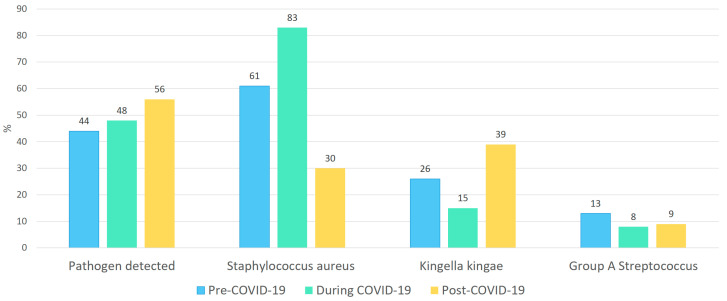
Distribution of pathogens in pOAI cases throughout the different phases. **Legend**: The most commonly identified pathogens were *Staphylococcus aureus*, in 51% of cases, followed by *Kingella kingae* in 27% and GAS in 10%. During the pandemic, the proportion of *Staphylococcus aureus* cases increased while *Kingella kingae* and GAS cases decreased. After the pandemic, there was a surge in *Kingella kingae* cases, whereas the proportion of Staphylococcus aureus reduced.

A causative pathogen from a sterile site was detected in 58/118 of patients (49%), including 28 from blood culture, 22 from joint aspirates, and 17 from bone punctures. This resulted in a total of 58/118 patients (49%) with a confirmed pathogen. *Staphylococcus aureus* was the most frequently identified organism (30/58, 51%), followed by *Kingella kingae* (16/58, 27%), GAS (6/58, 10%), and *Streptococcus pneumoniae* (4/58, 7%). Of the 16 *Kingella kingae* cases, 15 were identified via PCR on puncture material and one via culture. The percentage of positive *Kingella kingae* PCR results from puncture samples by year is shown in Table 3. Less frequently detected pathogens included *Neisseria meningitidis* and *Haemophilus influenzae*. Period-specific pathogen distribution is presented in Table 2 and Figure 2.

Between P1 and P2, the proportions of *Kingella kingae* and GAS decreased, while the prevalence of *Staphylococcus aureus* remained stable. In P3, the proportion of *Kingella kingae* increased, whereas *Staphylococcus aureus* showed a decrease, and GAS remained relatively unchanged. However, none of these changes were statistically significant (*p* > 0.05 for all comparisons).

## 4. Discussion

This 9-year retrospective study suggests an increase in pOAI at this center during the post-pandemic period (P3) compared to the pre-pandemic period (P1), while patient characteristics and clinical outcomes remained largely unchanged. During the pandemic period (P2), a non-significant decrease in *Kingella kingae* and GAS as causative pathogens was observed, accompanied by a relative increase in *Staphylococcus aureus*. In contrast, the post-pandemic period was characterized by a proportional rise in *Kingella kingae* detections, while the contribution of *Staphylococcus aureus* declined.

To our knowledge, no other studies to date (as of June 2025) have reported a general increase in pOAI following the COVID-19 pandemic. Pigeolet et al. described a post-pandemic rise in GAS osteoarticular infections in France, though this was pathogen-specific and not reflective of overall pOAI incidence [12]. It is interesting that a broader increase in pOAI has not been widely noted elsewhere. Considering that most pOAI cases arise through hematogenous spread, the resurgence of viral respiratory infections following the relaxation of NPIs may have facilitated bacterial hematogenous spread through viral-induced epithelial disruption [13]. Combined with the recent increases in bacterial infections and evidence of heightened post-pandemic susceptibility in children, these factors may contribute to the trends we observed [1,2,3,7,8,9,10,14]. This highlights an important area for further study. Furthermore, we found no published studies addressing changes in the distribution of pathogens causing pOAI across the pre, mid-, and post-pandemic periods. We hypothesize these possible changes are related to pathogens’ specific pathophysiologies. While *Staphylococcus aureus* typically colonizes the skin and mucosal surfaces and is known to have maintained stable colonization rates during the pandemic, *Kingella kingae* and GAS primarily colonize the oropharynx [15,16,17]. The implementation of NPIs, including school and daycare closures, likely reduced exposure and transmission of *Kingella kingae*, which is most prevalent among children attending early childcare facilities. This could explain the possible shifts we observed.

An apparent increase in abscess formation over the study period was also observed. While earlier years showed lower abscess rates (~10%), this may reflect underdiagnosis. Both Clemente et al. and Johnston et al. report that 40–45% of pOAI have abscess formation, mostly identified by MRI [18,19]. The observed increase in the proportion of patients presenting with abscesses over time may be partly influenced by evolving imaging practices. Although MRI use was highest during the earliest period, abscess detection increased in subsequent periods when MRI use was relatively lower, suggesting that other factors may also contribute. Nevertheless, improved imaging availability and more selective use of MRI in patients at higher risk for abscess likely influenced detection rates, introducing potential detection bias. This limitation should be considered when interpreting trends in abscess incidence.

### 4.1. Implications

The observed increase in pOAI cases in the post-pandemic period raises important clinical considerations. This possible epidemiological shift highlights the need for sustained clinical vigilance and systematic surveillance to monitor trends, identify emerging patterns, and guide diagnostic and therapeutic strategies. Strengthening clinical surveillance networks may enable earlier recognition of changing incidence rates, ultimately supporting more responsive and tailored care.

Additionally, the rising detection of abscesses over the study period has both diagnostic and management implications. While this trend is likely influenced by evolving imaging practices and possibly greater clinical awareness, a true change in disease severity or presentation cannot be ruled out. This underscores the importance of timely imaging—particularly MRI—in cases with atypical progression or elevated inflammatory markers, to promptly identify complications and optimize intervention.

Furthermore, with 50% of pOAI cases in our cohort being culture-negative and none requiring PICU admission or developing sepsis, our findings support reconsideration of empiric treatment approaches. In clinically stable, uncomplicated cases, early transition to oral antibiotic therapy—or even exclusive oral treatment—may be a safe and effective option. Recent evidence suggests that shorter and less invasive antibiotic regimens can achieve favorable outcomes in selected pediatric patients, provided there is close follow-up and adherence to established protocols [6,20,21]. These results reinforce the need to individualize treatment strategies based on clinical severity, pathogen identification, and host factors, potentially reducing the risks and resource demands associated with intravenous therapy and hospitalization.

### 4.2. Strengths and Limitations

This study has several important strengths. It provides a comprehensive nine-year analysis of pOAI spanning the pre-, mid-, and post-pandemic periods within a well-defined clinical setting. The consistent diagnostic protocols, including stable PCR testing methods since 2019, enhance the reliability of trend analyses. Additionally, clinical and microbiological data were systematically collected, allowing for nuanced insights into pathogen distribution. Finally, by capturing data from a large pediatric hospital that serves both local and regional populations, the study reflects a diverse patient cohort, strengthening the clinical relevance of the findings.

However, the study also has limitations. The single-center design limits the generalizability of our findings. As the study was retrospective in nature and based on routinely collected clinical data, a predefined power analysis was not performed. This means that some observed non-significant differences, such as changes in pathogen proportions, may reflect limited sample size rather than true absence of effect. Besides, the increased detection rate of *Kingella kingae* after 2017 largely reflects the introduction and broader implementation of PCR testing for this pathogen. This diagnostic advancement likely contributed to earlier observed increases in *Kingella kingae* proportions, representing an ascertainment bias rather than a true epidemiological change. However, since 2019, PCR testing has been steadily implemented and consistently applied, supporting that the post-2019 trends more accurately reflect true epidemiological changes rather than methodological artifacts. Another consideration is the potential influence of altered health-seeking behavior during the COVID-19 pandemic. While it is possible that hesitancy to seek hospital care or shifts in referral patterns impacted presentation rates, we anticipate that the severity and persistence of pOAI symptoms would still prompt medical evaluation. Nonetheless, these factors might possibly have affected the observed numbers and clinical characteristics during the pandemic period. Lastly, *Kingella kingae* infections might be underrepresented in our cohort because these cases often present with milder symptoms and diagnostic punctures may have been less frequently performed, limiting pathogen confirmation. Culture-negative cases represent a known challenge in pOAI, often representing uncomplicated cases where invasive sampling was either deemed unnecessary or unfeasible. In our cohort, these cases were classified according to established clinical and imaging criteria, with treatment decisions guided by standard protocols. Overall, these limitations underscore the need for multicenter studies to validate and expand upon our findings, assess broader epidemiological shifts in pOAI post-pandemic, and refine treatment approaches accordingly.

## 5. Conclusions

Based on a 9-year retrospective single-center cohort study, there are signs of an increase in pOAI after the COVID-19 pandemic. While patient characteristics and outcome remained similar over time, there appeared to be a change in pathogen distribution. These findings highlight the need for further multicenter research to assess whether this reflects a broader epidemiological change and to guide future diagnostic and therapeutic strategies).

## Figures and Tables

**Table 1:** Overview of criteria for diagnosis of osteomyelitis and arthritis, adapted from Trobisch et al. [4].

	Osteomyelitis	Arthritis
Confirmed cases	Bacteriologic evidence (positive blood and/or bone culture) plus at least one of the following: (1) localized pain or tenderness with other typical features such as warmth or swelling of the affected region; (2) imaging findings consistent with osteomyelitis (e.g., MRI or bone scan); or (3) histopathological confirmation (intraoperative specimen)	Isolation of a microorganism from blood or synovial fluid and imaging consistent with arthritis (MRI and/or bone scan).
Suspected cases	Localized pain or tenderness and other typical clinical features of osteomyelitis, such as warmth and/or swelling of the affected region; and imaging that excludes other diagnoses (X-ray and/or ultrasound); and followed an osteomyelitis treatment plan	Presence of localized pain/tenderness and other typical features of arthritis, such as warmth and/or swelling of the affected region; and image findings that are consistent with arthritis (ultrasound and/or X-ray); and followed an arthritis treatment plan
Rejected cases	No current presentation; or did not visit a pediatric or orthopedic doctor; or was not treated with an osteomyelitis treatment plan; or diagnosis or treatment was performed outside the Juliana Children’s Hospital; or image findings were not consistent with or exclude osteomyelitis (MRI, bone culture, X-ray, and/or ultrasound); or was autoimmune-related osteomyelitis (e.g., chronic recurrent multifocal osteomyelitis)	No current presentation; or did not visit a pediatric or orthopedic doctor; or was not treated with an arthritis treatment plan; or diagnosis or treatment was performed outside the Juliana Children’s Hospital; or image findings were not consistent with or exclude arthritis (MRI, bone culture, X-ray, and/or ultrasound); or was autoimmune-related arthritis (e.g., reactive arthritis or rheumatoid arthritis, including juvenile idiopathic arthritis and Henoch–Schönlein purpura arthritis)

**Table 2:** Characteristics of pOAI patients defined by in total and during the three COVID-19-related periods.

	Total N = 118 (%)	Pre-COVID-19 P1 (2015–2019) N = 52 (%)	Mid-COVID-19 P2 (2020–2021) N = 25 (%)	Post-COVID-19 P3 (2022–2023) N = 41 (%)
General characteristics				
Gender, female	46 (39)	18 (35)	10 (40)	18 (44)
Median age, in months	24 (IQR 12–96)	24 (IQR 10–87)	48 (IQR 24–96)	30 (IQR 12–96)
Arthritis	50 (42)	20 (39)	11 (44)	19 (46)
Osteomyelitis	59 (50)	28 (54)	13 (52)	18 (44)
Osteomyelitis and arthritis	9 (8)	4 (8)	1 (4)	4 (10)
Pathogen				
Pathogen detected	58 (49)	23 (44)	13 (48)	22 (54)
*Staphylococcus aureus*	30 (51)	14 (61)	8 (83)	8 (36)
Group A *Streptococcus*	6 (10)	3 (13)	1 (8)	2(9)
*Kingella kingae*	16 (27)	6 (26)	2 (15)	9 (40)
Other *	6 (10)			
Diagnostic findings at presentation				
CRP level determined at first presentation	117 (99)			
Median CRP level (mg/L)	60.5 (IQR 28.5–121)	61.5 (IQR 37–39)	41 (IQR 5–98)	75 (IQR 25–135.5)
ESR level determined at first presentation	93 (79)			
Median ESR level in mm	38 (IQR 19–60)	39 (IQR 24–52)	37 (IQR 15.5–67)	31 (IQR 19–70)
Abscess	23	5/51 (10)	7/25 (28)	11/41 (27)
Outcome				
Pediatric intensive care unit admission	0			
Mortality	0			

* Other detected pathogens included Streptococcus pneumonia, Neisseria meningitidis, and Haemophilus influenzae.

**Table 3:** Overview of the number of joint and bone punctures, number and percentage of PCR performed on *Kingella kingae*, and number and percentage of positive *Kingella kingae* cases.

	2015	2016	2017	2018	2019	2020	2021	2022	2023	Total
Number of joint and/or bone punctures per year	4	3	8	6	5	10	6	12	17	71
Number and % of PCR performed on *Kingella kingae* in bone and/or joint puncture	0	0	1 (12.5)	1 (16.6)	3 (60)	2 (20)	4 (66)	8 (66)	12 (70)	30 (42)
Number and percentage of positive PCR on *Kingella kingae*	0	0	1 (12.5%)	1 (16.6%)	3 (60%)	1 (10%)	1 (16.6%)	5 (41.6%)	3 (17.6%)	15 (21.1%)

## Data Availability

The data presented in this study are available upon reasonable request from the corresponding author. The data are not publicly available due to [privacy and ethical restrictions].

## Supplementary Materials

The following supporting information can be downloaded at: https://www.mdpi.com/article/10.3390/children12091210/s1, Supplementary content S1. Definitions Text.

## Abbreviations

The following abbreviations are used in this manuscript:

AR	septic or infectious arthritis
GAS	Group A streptococcus
NPI	non-pharmaceutical interventions
OA	osteomyelitis combined with arthritis
OM	osteomyelitis
