# Supplementary material for: Epidemiological Trends in Pediatric Osteoarticular Infections—Results from a Single-Center Retrospective Study Covering 2015–2023
Source: Children (Basel). 2025 Sep 10;12(9):1210. doi: 10.3390/children12091210 (PMC12468746; doi:10.3390/children12091210)
Supplement: Supplementary file 1 [file children-12-01210-s001.zip › children-3739637-supplementary.pdf]

Supplementary digital content 1. Definitions Text

Based on:

Trobisch A, Schweintzger NA, Kohlfürst DS et al; EUCLIDS consortium. Osteoarticular Infections in Pediatric Hospitals in Europe: A Prospective Cohort Study From the EUCLIDS Consortium. Front Pediatr. 2022 May 4;10:744182.

Diagnosed cases

Osteomyelitis diagnosis was based on the following criteria, of which at least two must be positive: bacteriologic evidence of infection (positive blood and/or bone culture); AND (1) Presence of localized pain/tenderness and other typical features of osteomyelitis such as warmth and/or swelling of the affected region. AND/OR (2) image findings consistent with osteomyelitis (typical MRI findings and/or positive bone scan) AND/OR (3) histopathological finding consistent with osteomyelitis (intraoperative specimen)

Arthritis was diagnosed when a microorganism was isolated from the blood or synovial fluid; AND image findings consistent with arthritis (typical MRI findings and/or positive bone scan).

Suspected cases

Osteomyelitis was diagnosed as suspected when; there was a presence of localized pain/tenderness and other typical features of osteomyelitis such as warmth and/or swelling of the affected region. AND image findings that exclude other diagnoses (X-ray photos and/or ultrasound); AND followed an osteomyelitis treatment plan.

Arthritis was diagnosed as suspected when there was a presence of localized pain/tenderness and other typical features of arthritis such as warmth and/or swelling of the affected region; AND image findings that are consistent with arthritis (ultrasound and/or X-ray); AND Followed an arthritis treatment plan.

Rejected cases

Osteomyelitis was rejected when it was treated in the past but no current presentation of osteomyelitis; OR did not visit a pediatric or orthopedic doctor; OR was not treated with an osteomyelitis treatment plan; OR Diagnosis or treatment was performed outside the Juliana Children's Hospital; OR image findings that are not consistent with or exclude osteomyelitis (MRI, bone culture, X-ray, and/or ultrasound); OR was autoimmune-related osteomyelitis (e.g. chronic recurrent multifocal osteomyelitis).

Arthritis was rejected when it was treated in the past but no current presentation of arthritis. ; OR did not visit a pediatric or orthopedic doctor; OR was not treated with an arthritis treatment plan; Diagnosis or treatment was performed outside the Juliana Children's Hospital; OR image findings that are not consistent with or exclude arthritis (MRI, bone culture, X-ray, and/or echo); OR was autoimmune-related arthritis (e.g. reactive arthritis or rheumatoid arthritis (including juvenile idiopathic arthritis) and Henoch-Schönlein purpura arthritis).

Histopathological finding

In osteomyelitis include the presence of inflammatory cells (in acute osteomyelitis: predominantly polymorph nuclear leucocytes, in chronic osteomyelitis: mononuclear cells including plasma cells and macrophage/monocyte cells) AND/OR destruction/necrosis of bone (necrotic marrow and bone,

osteoclastic activity) AND/OR granulation tissue (hemorrhage, polymorph nuclear leucocytes, lymphocytes, and macrophages).

Image findings consistent with osteomyelitis

MRI detecting and localization of the connective tissue and/or bone complications

Bone scan localization of infection

X-ray no visual fracture and/or malignancy that could suggest another cause of the clinical representation AND/OR osseous abnormalities (e.g. osteopenia, periosteal reaction/thickening, necrose cortex renal, loss of trabecular architecture, peripheral sclerosis, new bone deposition)

Ultrasound presence of abscess, cellulitis, and/or subperiosteal pus collection.

Image findings consistent with arthritis

MRI detecting and localization of the connective tissue and/or joint complications.

Bone scan localization of infection

X-ray no visual fracture and/or malignancy that could suggest another cause of the clinical representation AND/OR osseous abnormalities (e.g. osteomyelitis or tumor)

Ultrasound to detect the presence of fluids in joint and/or synovitis.
